# A Radium Policy for Poor-Law Unions

**Published:** 1914-03-21

**Authors:** 


### The Hospital

The Workers' Newspaper of Administrative Medicine and Institutional Life, Administration, National Insurance and Health.

No. 1447, Vol. LV. SATURDAY, MARCH 21, 1914. PRICE ONE PENNY.

### A RADIUM POLICY FOR POOR-LAW UNIONS.

The Special Number on Radium which we publish this week would be incomplete were not the problem which it offers to Poor-Law authorities specifically dealt with. The Poor-Law authorities are naturally awaiting the fuller experiences which the voluntary hospitals are beginning to give before following their example, but the question is already being actively canvassed in certain Unions, and therefore we have thought it well in this Special Number, which contains an expert summary of the knowledge at present available on the commercial, therapeutic, and institutional aspects of the question, to devote a leading article to the policy which the Poor-Law Unions should adopt in the light of the facts discussed elsewhere. The policy for the voluntary hospitals is becoming that of purchase wherever a fund can be raised to pay the cost, but in the Poor-Law world no policy has yet been decided on. Without such a policy the facts with which the voluntary hospitals are concerning themselves would lose much in value, and therefore the deductions which they offer to the Poor-Law authorities should be taken advantage of in framing a course in connection with what soon will be, if it is not at the moment, an equally important issue.

The recent advances which have been made in our knowledge of radium, and the striking results which have in some cases followed its use, have naturally caused many Poor-Law medical officers to try to obtain a supply for the treatment of their patients. In a few instances a small supply, obtained when prices were much lower than they are now or loaned by members of the medical staff or their friends, is already available. The high prices ruling to-day and the comparatively large amounts now considered necessary for effective use render any further provision impossible, except by the largest Boards of Guardians. It must be added that the results so far obtained, striking as they are in individual cases, are too conflicting to justify the enormous expense which would be entailed if all Poor-Law infirmaries were to be provided with an adequate supply. Nevertheless there are special reasons why Poor-Law medical officers should be anxiously watching the trend of research in radium therapy and should be prepared, if the hopes freely expressed by enthusiastic workers on the subject are justified by fuller experience, to press for some provision being made. Further investigations and observations may disappoint or mitigate our hopes, but it does seem as if we may soon be in a position to assert that we possess in radium a means of greatly ameliorating certain cases of malignant disease, such as, for instance, rheumatic arthritis, chronic gout, Bright's disease, chronic bronchitis, and chronic skin diseases—all of them diseases for which our present methods of treatment are notoriously inadequate. Now it is just these diseases in their worst, most crippling, and most recalcitrant forms from which a large proportion of the inmates of our Poor-Law infirmaries and workhouses suffer. Patients afflicted with all these diseases, having exhausted their resources or been pronounced incurable by their medical attendants or by the voluntary hospitals, drift finally to the Poor Law, to spend the remainder of their lives within the walls of its institutions.

Exact figures are not obtainable; but one may deduce from the figures given in the Registrar-General's return that in England and Wales about 4,000 patients, and in London about 1,200, die every year in Poor-Law institutions from some form of malignant disease. As regards gout and rheumatic conditions, a return obtained by the Local Government Board showed that on November 4, 1911, there were nearly 13,000 cases in the Poor-Law institutions of England and Wales. From 50 to 60 per cent. of these would be chronic cases, more or less permanently incapacitated. Add to these the still larger figures of incapacity due to chronic bronchitis and chronic Bright's disease, and some idea can be formed of the magnitude of the problem and of the immense saving in pain and in the material wealth of the community which would result if radium should really prove to combat the ravages of these complaints. One may say at once that if radium should be firmly established as a curative agent for these conditions—in the same sense as one can speak of mercury and salvarsan as curative agents in syphilis—then the initial expense would be more than justified and would result in the end in an immense saving. But it is just here that doubt creeps in and we feel compelled to advise caution before embarking on large and expensive schemes. Remarkable as the results sometimes are, for instance, in malignant disease, no one has yet ventured to suggest that, apart from rodent ulcer, cancer, in a part of the body easily accessible to the surgeon's knife, should be treated by radium in preference to operation. That fact in itself, when one remembers the large number of cases in which operative treatment fails, should teach caution. Nor can one shut one's eyes to those cases in which the malignant growth has seemed to be stimulated rather than retarded by the exhibition of radium.

As a practical beginning the Unions in large areas, such as London and the greater provincial towns, should unite to establish in each area a radium institute equipped with a moderate amount of radium under the charge of a medical man with special knowledge of radium and its applications. Radium emanation would be prepared in these institutes and supplied to the various infirmaries in the areas of the combined Unions for use in cases carefully selected as being most likely to benefit by the treatment. Similarly, cases would be carefully picked out for treatment by radium itself. The tubes and applicators would be lent from the institute, and would be applied in accordance with the advice of the director of the institute. In this way the maximum benefit would be obtained at a comparatively moderate expenditure, and the amount of material available could be increased and the range of cases treated extended as increased knowledge and experience justified the additional expenditure. We would lay stress, however, upon the necessity of providing an expert in radium therapy as well as the radium itself if the best results are to be obtained. Past experience shows that Guardians will often spend money freely in providing institutions and equipment, but fail to realise the still greater importance of providing the skilled staff necessary to make the best use of the means provided. Radium in unskilled hands is more likely to prove a danger than a help to the patient, and the medical superintendent of an infirmary cannot nowadays be an expert in all the specialised branches of medical work.

